# Supplementary material for: GATA3 induces mitochondrial biogenesis in primary human CD4+ T cells during DNA damage
Source: Nat Commun. 2021 Jun 7;12:3379. doi: 10.1038/s41467-021-23715-7 (PMC8184923; doi:10.1038/s41467-021-23715-7)
Supplement: Supplementary file 1 — Supplementary Information [file 41467_2021_23715_MOESM1_ESM.pdf]

GATA3 induces mitochondrial biogenesis in primary human CD4^{+} T cells during DNA damage

Lauren A. Callender, Johannes Schroth, Elizabeth C. Carroll, Conor Garrod-Ketchley, Lisa E.L. Romano, Eleanor Hendy, Audrey Kelly, Paul Lavender, Arne N. Akbar, J. Paul Chapple, and Sian M. Henson

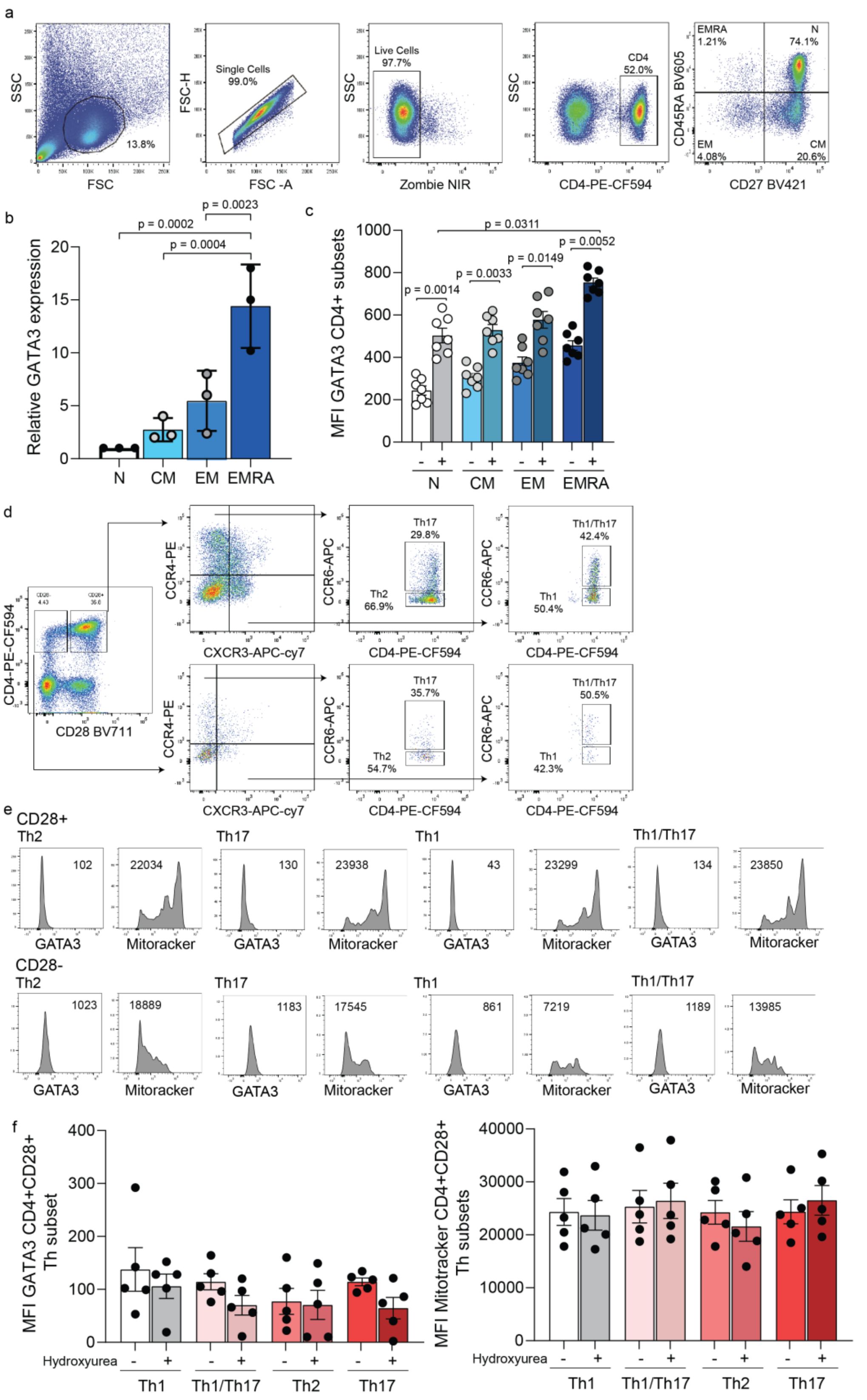

Supplementary Figure 1: Expression of GATA3 in CD4+ T cell defined by differentiation and Th function. (a) Gating strategy for CD27/CD45RA defined CD4+ T cell subsets. From left to right, lymphocytes, single cells, live cells, CD4+ T cells and CD27/CD45RA subsets. (b) qPCR data showing the ΔCT relative expression of GATA3 in CD4+ T cell subsets (n=3 biologically independent samples). (c) GATA3 staining in CD27/CD45RA defined CD4+ T cell subsets with and without overnight stimulation with 0.5 μg/ml anti-CD3 (n=7 biologically independent samples). (d) Gating strategy for CD28/Th defined CD4+ T cells. From left to right, CD4+CD28+/- T cells, CCR4/CXCR3 T cells, CCR4+CXCR3- T cells followed by CCR6/CD4 gives rise to Th2 and Th17 T cells, CCR4-CXCR3+ T cells followed by CCR6/CD4 give rise to Th1 and Th1/Th17 T cells. (e) Representative staining of GATA3 and mitotracker green in CD4+ Th subsets defined above. (f) GATA3 and mitotracker green staining in CD4+CD28+ Th defined subsets (n=5 biologically independent samples for both graphs). P values were determined using a Kruskal Wallis followed by Dunn multiple comparison for post-hoc testing. Graphs show ± SEM.

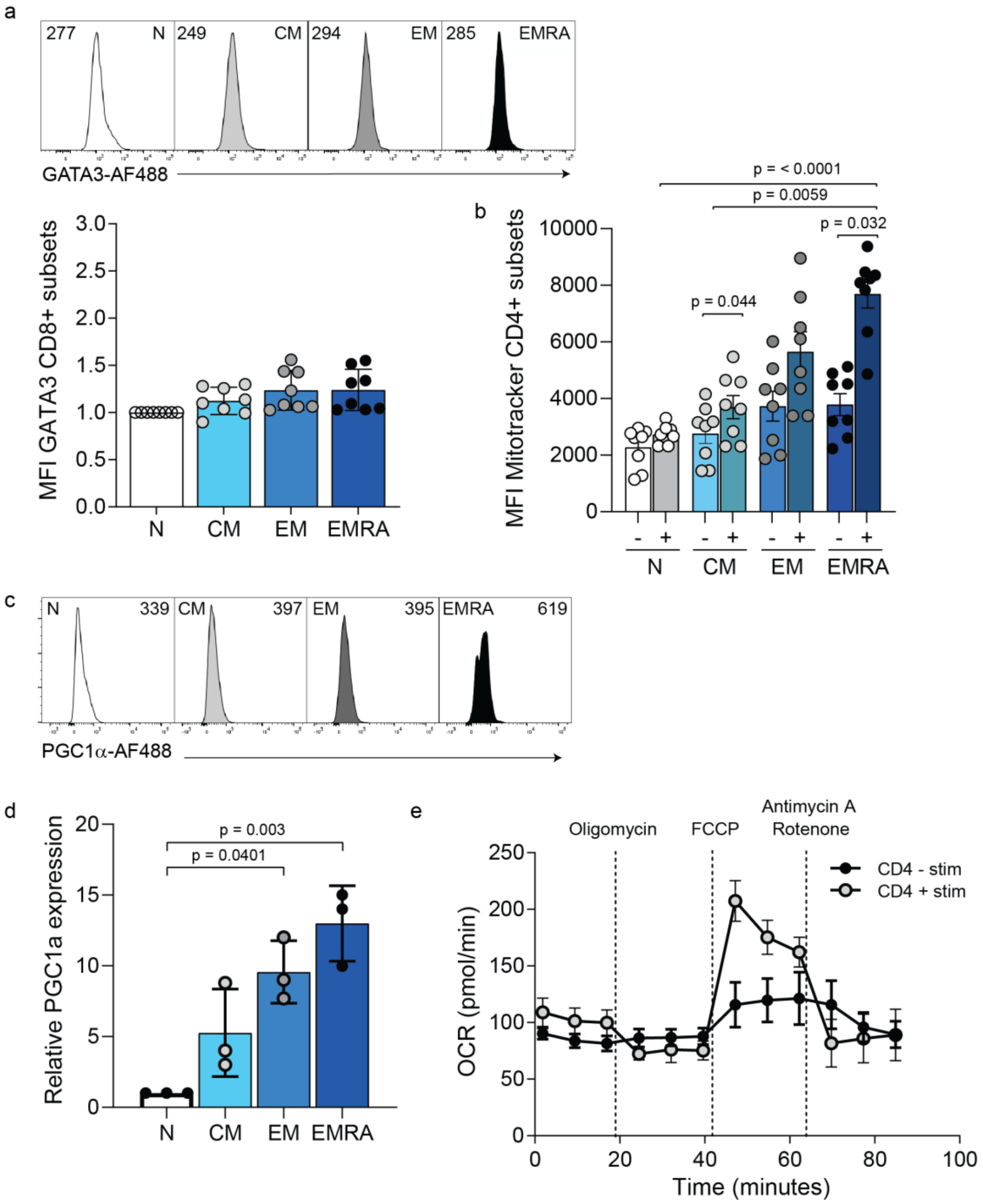

Supplementary Figure 2: Expression of GATA3 in CD4+ T cell subsets

(a) GATA3 expression in unstimulated CD27/CD45RA defined CD8+ T cell subsets (n=8 biologically independent samples). (b) Mitochondrial mass measured by mitotracker green staining in CD27/CD45RA defined CD4+ T cell subsets with and without overnight stimulation as described above (n=8 biologically independent samples). (c) Representative flow cytometry histograms of PGC1α staining in CD27/CD45RA defined CD4+ T cell subsets (n=8 biologically independent samples). (d) qPCR data showing the ΔCT relative expression of PGC1α in CD4+ T cell subsets (n=3 biologically independent samples). (e) OCR of the CD4+ T cells with and without 15 minute stimulation with 0.5 μg/ml anti-CD3 and 5 ng/ml IL-2, cells were subjected to a mitochondrial stress test using indicated mitochondrial inhibitors. Data is representative of 3 biologically independent samples. P values were determined using a Kruskal Wallis test followed by Dunn multiple comparison for post-hoc testing. Graphs show ± SEM.

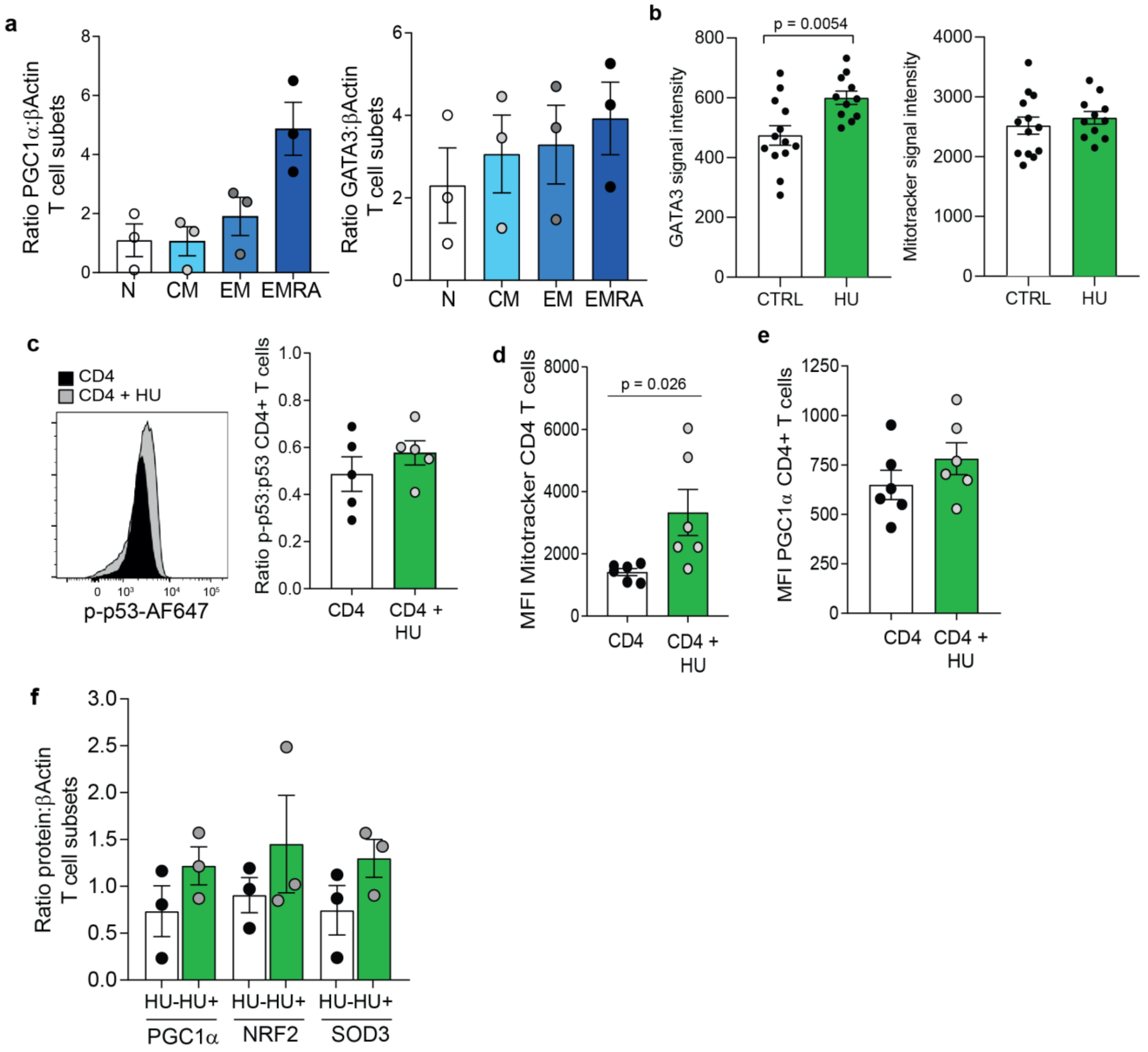

Supplementary Figure 3: Expression of GATA3 following hydroxyurea treatment

(a) Quantification of PGC1α and GATA3 following immunoprecipitation, shown as the ratio of PGC1α:βActin and GATA3:βActin (n=3 biologically independent samples). (b) Quantification of GATA3 and mitochondrial mass by signal intensity from confocal data (n=13 cells measured from 3 different individuals). (c) Stain of p-p53 and the p53:p-p53 ratio following the induction of DNA damage by 400 μM hydroxyurea in whole CD4+ T cells. Mitotracker Green staining (d) and PGC1α (e) in CD4+ T cells treated with and without (n=6 biologically independent samples). (e) Quantification of GATA3 and mitochondrial mass by signal intensity from confocal data (n=3 biologically independent samples). (f) Quantification of PGC1α NRF1 and SOD3 following immunoprecipitation, shown as the ratio:βActin (n=3 biologically independent samples). P values were determined using a two-way Mann-Whitney U test. Graphs show ± SEM.

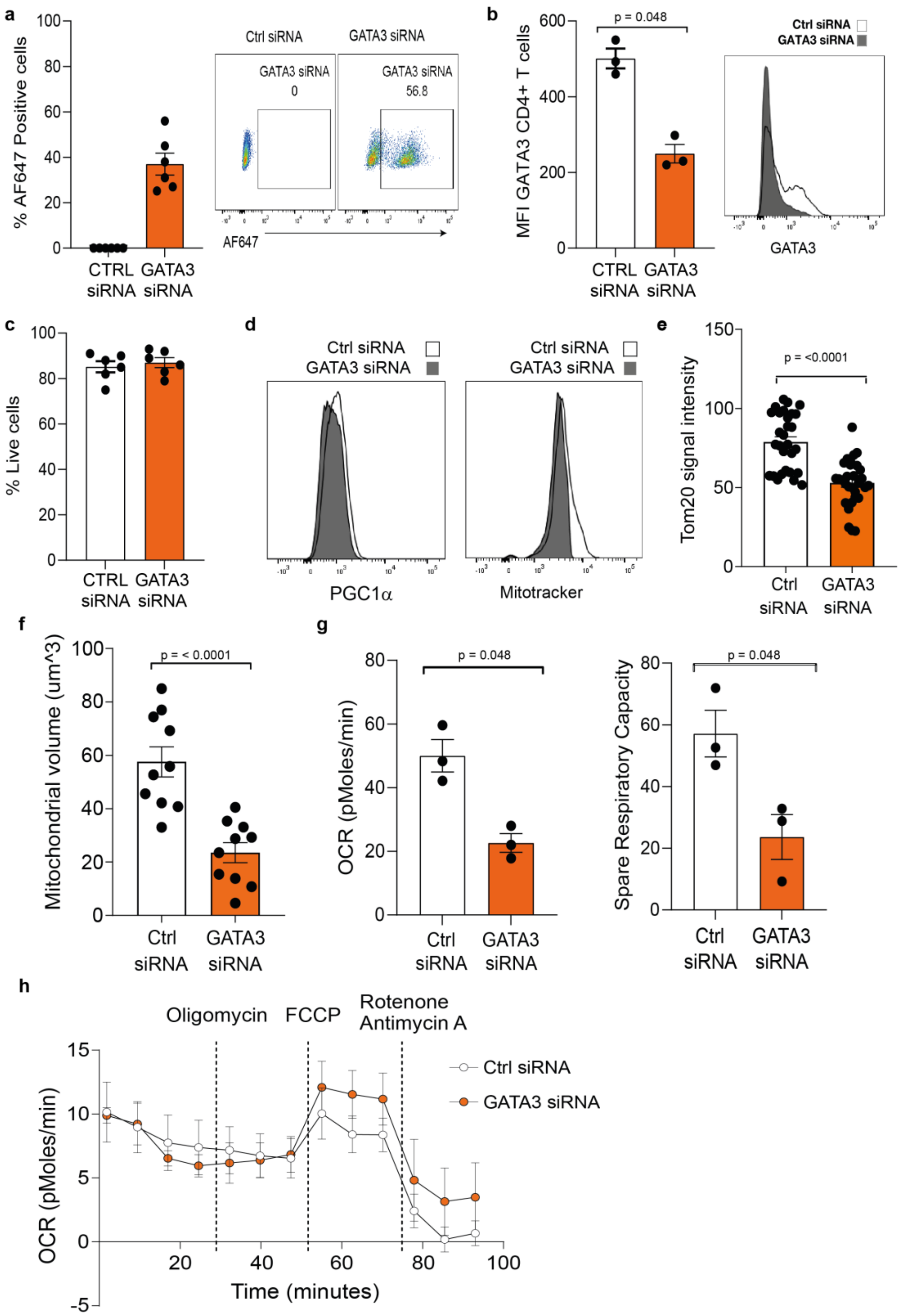

Supplementary Figure 4: siRNA knockdown of GATA3
(a) GATA3 siRNA transfection efficiency (n=6 biologically independent samples). (b) GATA3 staining following GATA3 siRNA transfection (n=3 biologically independent samples). (c) Percentage of live cells determined by Zombie NIR staining following GATA3 siRNA transfection (n=6 biologically independent samples). (d) Examples of PGC1α expression and mitochondrial mass following siRNA transfection. (e) Tom20 confocal signal intensity in Jurkat T cells transfected with either control or GATA3 siRNA (n=30 images). (f) Mitochondrial volume analysis from confocal microscopy data following GATA3 siRNA transfection (n=10 images). (g) Basal OCR and SRC of the CD4+ siRNA transfected cells measured after 15 minute stimulation with 0.5 μg/ml anti-CD3 and 5 ng/ml IL-2 by XF flux analysis (n=36 repeated measures from 3 individuals). (h) OCR of a mitochondrial stress test using indicated mitochondrial inhibitors from whole CD8+ T cells transfected with either control or GATA3 siRNA and stimulated as described above (n=3). P values were determined using a one-way Mann-Whitney U test for part b and g and a two-way Mann-Whitney U test was used for parts e and f. Graphs show ± SEM.

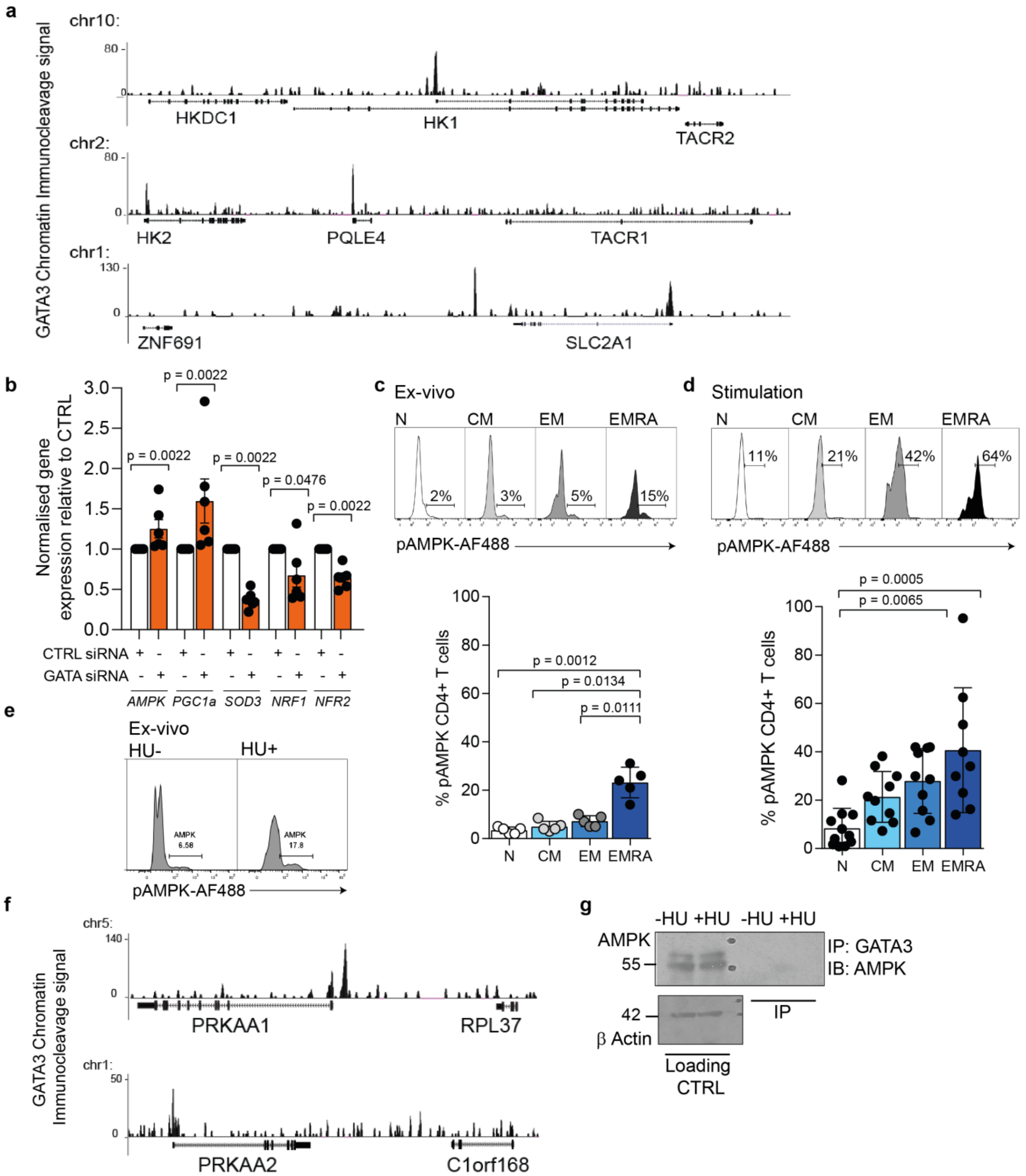

Supplementary Figure 5. Chromatin immunocleavage of the GATA3 promoter region

(a) Chromatin immunocleavage showing GATA3 binding to the promoter regions of hexokinase 1/2 (hk1) and SLC2A1 (slc2a1) in naïve CD4+ T cells. (b) qPCR data shown the levels of mRNA expression of PGC1a, AMPK, SOD3, NRF1 and NRF2 in Jurkat T cells following siRNA transfection with either the scrambled control or GATA3-AF647 siRNA after overnight treatment with 400 µM Hydroxyurea (n=3 biologically independent samples). (c) Representative examples and graphs of pAMPK (Thr172) staining in CD27/CD45RA defined CD4+ T cells ex-vivo (n=5 biologically independent samples) and after overnight stimulation (d) with 0.5 ug/ml aCD3 (n=9 biologically independent samples). (e) Example of pAMPK staining in CD4+ T cells following overnight incubation with 400 µM hydroxyurea. (f) Chromatin immunocleavage showing GATA3 binding to the promoter regions of AMPKα1 (PRKAA1) and AMPKα2 (PRKAA2) in naïve CD4+ T cells. (g) Western blot showing both the loading control and GATA3 immunoprecipitation in CD4+ T cells quantifying the presence of AMPK and βActin after hydroxyurea treatment (n=3 biologically independent samples). P values were determined using a Kruskal Wallis test followed by Dunn multiple comparison for post-hoc testing. Graphs show ± SEM.

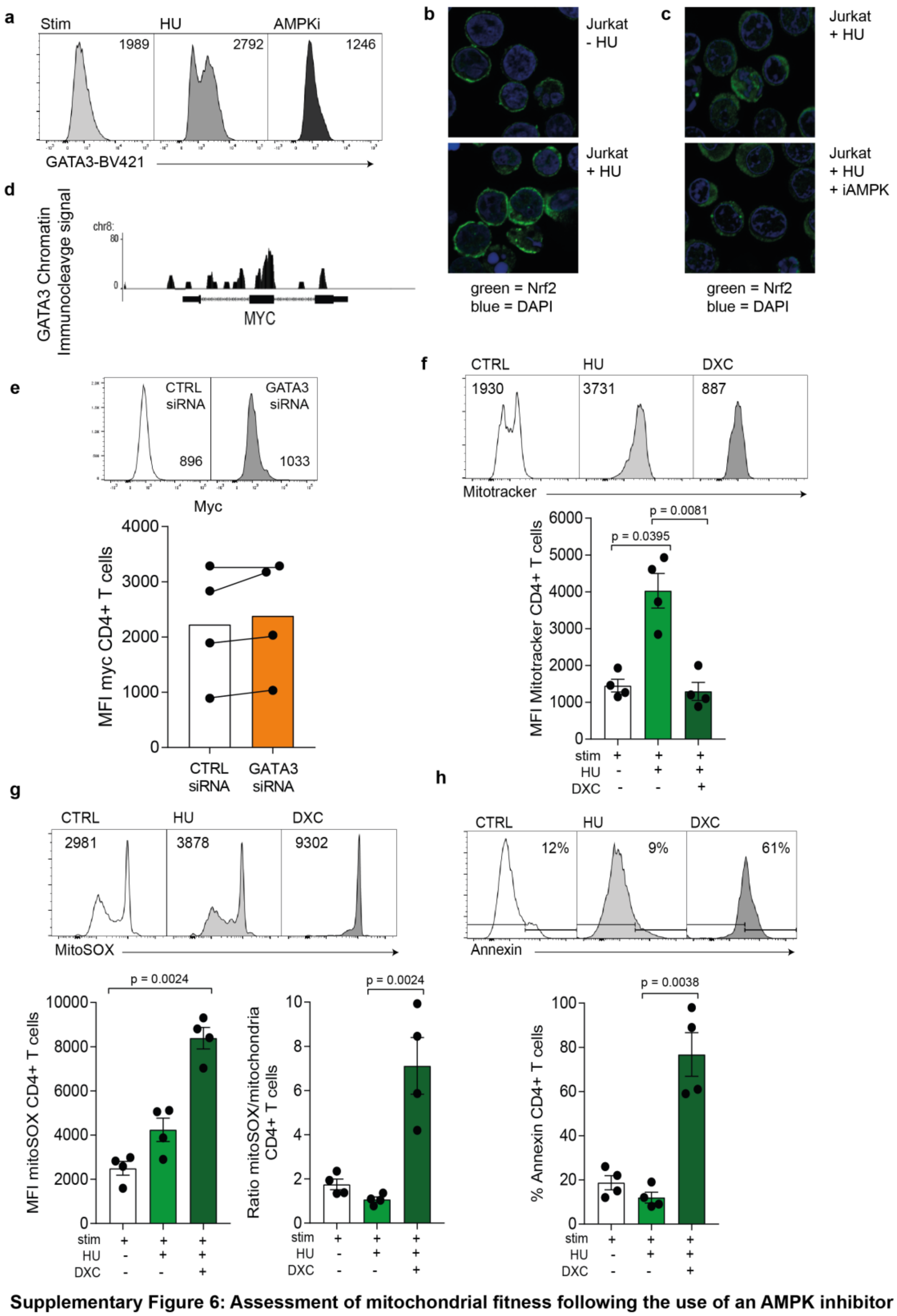

Supplementary Figure 6: Assessment of mitochondrial fitness following the use of an AMPK inhibitor and doxycycline.

(a) Example of GATA3 staining in CD4+ T cells following overnight treatment with 400 μM hydroxyurea and 1 μM AZD6738 (b) Nrf2 confocal staining and signal intensity data in Jurkat T cells following hydroxyurea treatment x63 (n=4 biologically independent samples). (c) Nrf2 confocal staining in Jurkat T cells following hydroxyurea treatment and 10 μM Compound C x63 (n=4 biologically independent samples). (d) Chromatin immunocleavage showing GATA3 binding to the promoter regions of c-myc (myc). (e) Data showing c-myc expression in CD4+ T cells following transfecting with either a control or GATA3 siRNA (n=4 biologically independent samples). (f) Representative examples and quantification of mitochondrial mass measured using mitotracker green, and (g) assessment of ROS production using mitoSOX in CD4+ T cells (n=4 biologically independent samples) following overnight incubation with hydroxyurea and 50 μM doxycycline.

(g) Representative examples of annexin V staining and assessment of apoptosis following treatment of CD4+ T cells with and without hydroxyurea and doxycycline (n=4 biologically independent samples). P values were determined using a two-way Mann-Whitney U test (part e) and a Kruskal Wallis test followed by Dunn multiple comparison for post-hoc testing. Graphs show ± SEM.

Supplementary Table 1. List of all primers used

Gene product	Gene name	Forward primer	Reverse primer
GATA3	*GATA3*	GCC CCT CAT TAA GCC CAA G	TTG TGG TCT GAC AGT TCG
PGC1α	*PPARGC1A*	TCT GAG TCT GTA TGG AGT GAC AT	CCA AGT CGT TCA CAT CTA GTT CA
AMPK	*PRKAA2*	TTG AAA CCT GAA AAT GTC CTG CT	GGT GAG CCA CAA CTT GTT CTT
SOD3	*SOD3*	ATG CTG GCG CTA CTG TGT TC	CTC CGC CGA GTC AGA GTT
NRF1	*NRF1*	GCT GAT GAA GAC TCG CTT TCT	TAC ATG AGG CCG TTT CCG TTT
NRF2	*NFE2L2*	TTC CCG GTC ACA TCG AGA G	TCC TGT TGC ATA CCG TCT AAA TC
BActin	*ACTB*	CAC CAT TGG CAA TGA GCG GTT C	AGG TCT TTG CGG ATG TCC ACG T
