# Supplementary material for: GATA3 induces mitochondrial biogenesis in primary human CD4+ T cells during DNA damage
Source: Nat Commun. 2021 Jun 7;12:3379. doi: 10.1038/s41467-021-23715-7 (PMC8184923; doi:10.1038/s41467-021-23715-7)
Supplement: Supplementary file 3 — Reporting Summary [file 41467_2021_23715_MOESM3_ESM.pdf]

nature research

Corresponding author(s): Sian Henson
Last updated by author(s): 09/04/2021

Reporting Summary

Nature Research wishes to improve the reproducibility of the work that we publish. This form provides structure for consistency and transparency in reporting. For further information on Nature Research policies, see Authors & Referees and the Editorial Policy Checklist .

Statistics

For all statistical analyses, confirm that the following items are present in the figure legend, table legend, main text, or Methods section.

n/a	Confirmed	
☐	☒	The exact sample size (n) for each experimental group/condition, given as a discrete number and unit of measurement
☐	☒	A statement on whether measurements were taken from distinct samples or whether the same sample was measured repeatedly
☐	☒	The statistical test(s) used AND whether they are one- or two-sided<br>*Only common tests should be described solely by name; describe more complex techniques in the Methods section.*
☒	☐	A description of all covariates tested
☐	☒	A description of any assumptions or corrections, such as tests of normality and adjustment for multiple comparisons
☐	☒	A full description of the statistical parameters including central tendency (e.g. means) or other basic estimates (e.g. regression coefficient) AND variation (e.g. standard deviation) or associated estimates of uncertainty (e.g. confidence intervals)
☐	☒	For null hypothesis testing, the test statistic (e.g. F, t, r) with confidence intervals, effect sizes, degrees of freedom and P value noted<br>*Give P values as exact values whenever suitable.*
☒	☐	For Bayesian analysis, information on the choice of priors and Markov chain Monte Carlo settings
☒	☐	For hierarchical and complex designs, identification of the appropriate level for tests and full reporting of outcomes
☐	☒	Estimates of effect sizes (e.g. Cohen's d, Pearson's r), indicating how they were calculated

Our web collection on statistics for biologists contains articles on many of the points above.

Software and code

Policy information about availability of computer code

Data collection	Flow cytometry samples were acquired on a LSR Fortessa flow cytometer (BD Biosciences); cells were scanned on a Zeiss LSM 880 confocal microscope and images were obtained using Z-stacks with a step-size of 0.5µm. For the electron microscopy the cells were viewed in a Jeol 1010 transmission electron microscope (Jeol) and imaged using a Gatan Orius CCD camera (Gatan). Metabolic data was collected using a Seahorse XFe96 Analyser (Agilent).
Data analysis	Flow cytometry data analysed using FlowJo software v10.7.1 (TreeStar). Surface rendered 3D images generated using the Surpass module of the Imaris image analysis software (Imaris 7.6.1 Bitplane) were then used to calculate mitochondrial number and volume. Protein bands in western blots were quantified using ImageJ software. Statistical analysis was performed using Prism v9 (GraphPad Software).

For manuscripts utilizing custom algorithms or software that are central to the research but not yet described in published literature, software must be made available to editors/reviewers. We strongly encourage code deposition in a community repository (e.g. GitHub). See the Nature Research guidelines for submitting code & software for further information.

Data

Policy information about availability of data

All manuscripts must include a data availability statement. This statement should provide the following information, where applicable:

- Accession codes, unique identifiers, or web links for publicly available datasets
- A list of figures that have associated raw data
- A description of any restrictions on data availability

The data that support the findings of this study are available from the corresponding author upon reasonable request. The raw numbers for charts and graphs are available in the Source Data file whenever possible, chromatin immunocleavge data has been deposited in the GEO database accession number GSE172346, https://www.ncbi.nlm.nih.gov/geo/query/acc.cgi?acc=GSE172346.

Field-specific reporting

Please select the one below that is the best fit for your research. If you are not sure, read the appropriate sections before making your selection.

☒ Life sciences ☐ Behavioural & social sciences ☐ Ecological, evolutionary & environmental sciences

For a reference copy of the document with all sections, see nature.com/documents/nr-reporting-summary-flat.pdf

Life sciences study design

All studies must disclose on these points even when the disclosure is negative.

Sample size	Samples sizes were different for each experiment according to availability of samples. Sample size is stated for each experiment.
Data exclusions	No data was excluded.
Replication	Experiments were repeated at least 3 times for reproducibility of data.
Randomization	This study was not a randomized control study.
Blinding	Blinding was not relevant to this study.

Reporting for specific materials, systems and methods

We require information from authors about some types of materials, experimental systems and methods used in many studies. Here, indicate whether each material, system or method listed is relevant to your study. If you are not sure if a list item applies to your research, read the appropriate section before selecting a response.

Materials & experimental systems

n/a	Involved in the study	
☐	☒	Antibodies
☐	☒	Eukaryotic cell lines
☒	☐	Palaeontology
☒	☐	Animals and other organisms
☐	☒	Human research participants
☒	☐	Clinical data

Methods

n/a	Involved in the study	
☒	☐	ChIP-seq
☐	☒	Flow cytometry
☒	☐	MRI-based neuroimaging

Antibodies

Antibodies used

BD Bioscience:
CD4 PE-CF594 - clone, RPA-T4; cat#, 562281
Biolegend:
CD45RA BV605 - clone, HI100; cat#, 304134. CD27 BV421 - clone, O323, cat#, 302824. GATA3 AF488 - clone, 16E10A23; cat#, 653808. γH2AX AF488 - clone, 2F3; cat#, 613406. Annexin cat# 640914; CCR4 PE - clone L291H4, cat# 359412; CCR6 APC - clone G034E3, cat# 353416; CXCR3 APC-cy7 - clone, G025H7, cat# 353722.
Cell Signaling Technology:
PGC1α - clone, 3G6; cat# 2178S. p53 Alexa Fluor 647 - clone 1C12; cat#, 2533S. p-p53 Alexa Fluor 647 (Ser15) - clone, 16G8; cat#, 8695S. pAMPK - clone, 40H9; cat# 2535S. βactin - clone 13E5; cat# 4970S.
Abcam:
goat anti-rabbit IgG H&L Alexa Fluor 488 - clone, polyclonal; cat#, ab150077. Chk1 - clone, E250; cat#, ab32531.

Validation

Validation was performed by the company, see website for details. All antibodies were optimized and titrated with appropriate positive and negative controls (isotype controls).

Eukaryotic cell lines

Policy information about cell lines

Cell line source(s)	Jurkat, Clone E6-1 cat# ATCC TIB-152
Authentication	The cells were authenticated by the ATCC at the point of purchase.

Mycoplasma contamination	We regularly check our cell lines for Mycoplasma contamination and they were negative.
Commonly misidentified lines (See ICLAC register)	*Name any commonly misidentified cell lines used in the study and provide a rationale for their use.*

Human research participants

Policy information about studies involving human research participants

Population characteristics	Healthy human participants aged over 18 (n=30 mean 41 years ± 5)
Recruitment	Healthy volunteers were recruited from recruitment drives of healthy volunteers (with no gender or age discrimination) wishing to participate in research studies. Participants were excluded if they had an infection or immunisation within the last month, they had to have no known immunodeficiency, and not to be receiving any immunosuppressive medications within the last 6 months.
Ethics oversight	The NRES Committee North East (REC reference: 16/NE/0073). All volunteers provided written, informed consent.

Note that full information on the approval of the study protocol must also be provided in the manuscript.

Flow Cytometry

Plots

Confirm that:

- [x] The axis labels state the marker and fluorochrome used (e.g. CD4-FITC).
- [x] The axis scales are clearly visible. Include numbers along axes only for bottom left plot of group (a 'group' is an analysis of identical markers).
- [x] All plots are contour plots with outliers or pseudocolor plots.
- [x] A numerical value for number of cells or percentage (with statistics) is provided.

Methodology

Sample preparation	Heparinized peripheral blood samples were taken from healthy volunteers. Peripheral blood mononuclear cells (PBMCs) were isolated using Ficoll-Paque (Amersham Biosciences). 1 x 106 PBMCs were used for flow cytometry and mitochondrial measurements.
Instrument	LSR Fortessa (BD Biosciences)
Software	FlowJo V10 was used to analyse the data, along with Prism v9.
Cell population abundance	Sorted CD27/CD45RA subsets were aquired using an ARIA FACS sorter (BD Bioscience), typically 40% of the PBMCs are CD4 T cells, however the amount of each subset varies per individual
Gating strategy	Gating strategy for CD27/CD45RA defined CD4+ T cell subsets: Lymphocytes, single cells, live cells, CD4+ T cells and CD27/CD45RA subsets.

- [x] Tick this box to confirm that a figure exemplifying the gating strategy is provided in the Supplementary Information.
